# Potential role of extracellular granzyme B in wet age-related macular degeneration and fuchs endothelial corneal dystrophy

**DOI:** 10.3389/fphar.2022.980742

**Published:** 2022-09-20

**Authors:** Eden Dubchak, Gideon Obasanmi, Matthew R. Zeglinski, David J. Granville, Sonia N. Yeung, Joanne A. Matsubara

**Affiliations:** ^1^ Department of Ophthalmology and Visual Sciences, University of British Columbia (UBC), Vancouver, BC, Canada; ^2^ ICORD Centre and Department of Pathology and Laboratory Medicine, Vancouver Coastal Health Research Institute, UBC, Vancouver, BC, Canada

**Keywords:** extracellular matrix, corneal endothelium, retinal pigment epithelium, serine protease, Descemet’s membrane, Bruch’s membrane

## Abstract

Age-related ocular diseases are the leading cause of blindness in developed countries and constitute a sizable socioeconomic burden worldwide. Age-related macular degeneration (AMD) and Fuchs endothelial corneal dystrophy (FECD) are some of the most common age-related diseases of the retina and cornea, respectively. AMD is characterized by a breakdown of the retinal pigment epithelial monolayer, which maintains retinal homeostasis, leading to retinal degeneration, while FECD is characterized by degeneration of the corneal endothelial monolayer, which maintains corneal hydration status, leading to corneal edema. Both AMD and FECD pathogenesis are characterized by disorganized local extracellular matrix (ECM) and toxic protein deposits, with both processes linked to aberrant protease activity. Granzyme B (GrB) is a serine protease traditionally known for immune-mediated initiation of apoptosis; however, it is now recognized that GrB is expressed by a variety of immune and non-immune cells and aberrant extracellular localization of GrB substantially contributes to various age-related pathologies through dysregulated cleavage of ECM, tight junction, and adherens junction proteins. Despite growing recognition of GrB involvement in multiple age-related pathologies, its role in AMD and FECD remains poorly understood. This review summarizes the pathophysiology of, and similarities between AMD and FECD, outlines the current knowledge of the role of GrB in AMD and FECD, as well as hypothesizes putative contributions of GrB to AMD and FECD pathogenesis and highlights the therapeutic potential of pharmacologically inhibiting GrB as an adjunctive treatment for AMD and FECD.

## Introduction

The leading causes of blindness in developed countries are primarily age-related ocular diseases, which constitute a sizable socioeconomic burden. Worldwide, visual impairments due to eye conditions (including age-related macular degeneration [AMD] diabetic retinopathy, cataract, uncorrected refractive error and other causes) incur an estimated annual cost of 2.302 trillion dollars US in direct health system costs, with another 625 billion dollars in indirect costs and loss of productivity (Gordois et al., 2012; Marques et al., 2022). Furthermore, the prevalence of eye diseases will continue to grow due to the aging population. In the United States, the prevalence of blindness (defined as visual acuity of 20/200 or less) among those between 40–69 years-of-age is estimated to increase by ∼23%, while for those in the age categories of 70–79 and 80+ is estimated to increase by 44 and 150%, respectively, from 2020 to 2050 (Varma et al., 2016).

**FIGURE 1:**
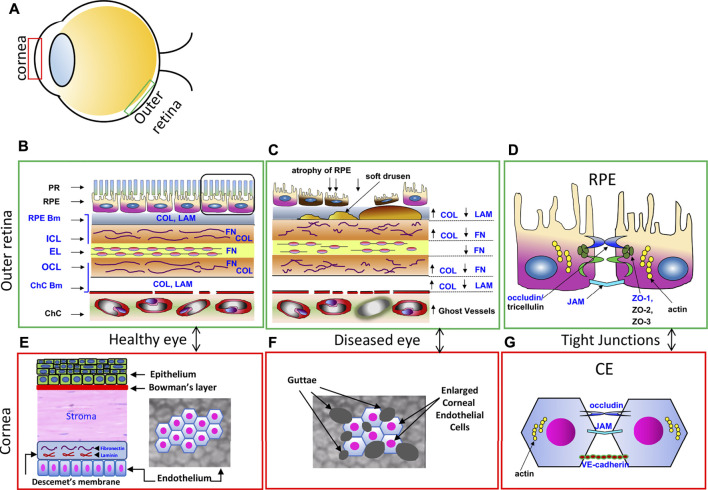
Comparisons of the RPE Monolayer and the Corneal Endothelial Monolayer. **(A)** Schematic diagram showing the location of the cornea and outer retina. **(B)** Enlarged diagram of the healthy outer retina in cross section, with photoreceptor outer segments (PR), retinal pigment epithelium (RPE) and underlying Bruch’s Membrane, an extracellular matrix that includes the RPE basement membrane (RPE Bm), the internal collagenous layer (ICL), elastic layer (EL) and outer collagenous layer (OCL) and the choroicapillaris (ChC) basement membrane (ChC bm). These layers contain collagen (Col), laminin (LAM), fibronectin (FN), substrates of GrB. Adapted from Matsubara et al., 2020. **(C)** In the AMD diseased eye, the ECM is disorganized; COL, FN and LAM are cleaved by GrB, causing a redistribution of ECM proteins. Soft drusen deposits develop, causing RPE atrophy and eventually cell death. **(D)** Tight junctional proteins between RPE support the outer blood eye barrier in the healthy eye. ZO-1, occludin, and JAM are substrates of GrB and are susceptible to GrB cleavage. **(E)** Enlarged diagram of the healthy corneal epithelium, stoma and corneal endothelium (CE) in cross section (left) and in flatmount views (right). Descemet’s membrane, an ECM, contains FN and LAM. **(F)** In the FECD diseased eye, the CE are enlarged with guttae deposits shown in the flatmount view. **(G)** Schematic diagram of the junctional proteins, occludin, JAM, and VE-cadherin, between CEC support the barrier function.

AMD is one of the most common age-related diseases that affects the retina and comprises 12% of the total global economic burden caused by visual impairment (Gordois et al., 2012). Fuchs endothelial corneal dystrophy (FECD) is one of the most common age-related diseases of the cornea and is the leading non-iatrogenic indication for corneal transplants worldwide (Gain et al., 2016). These two diseases, while seemingly disparate and located in retina and cornea (Figure 1A), are characterized by the degeneration of a monolayer of post-mitotic cells with important cellular barrier functions. During the development of AMD, the retinal pigment epithelial (RPE) monolayer undergoes degenerative changes due to several factors such as oxidative stress, complement cascade attack and chronic inflammation that promote cellular dysfunction and atrophy (Datta et al., 2017). Similarly, during the development of FECD, the corneal endothelial monolayer (CE) undergoes degenerative changes due to factors including oxidative stress, mitochondrial dysfunction and endoplasmic reticulum (ER) stress. These changes lead to significant alterations in gene expression patterns, culminating in accelerated atrophy of the RPE and the corneal endothelial cells (CEC) in AMD and FECD respectively (Bergmanson et al., 1999; Ambati and Fowler, 2012; Lin et al., 2013; Ong Tone et al., 2021). A major aspect of the pathogenesis of both AMD and FECD is the disorganization of the local extracellular matrix (ECM) and the toxic accumulation of protein deposits (referred to as drusen in AMD and guttae in FECD) on the underlying basement membrane (Bruch’s membrane in AMD and Descemet’s membrane in FECD). These pathogenic mechanisms can be linked to both altered expression patterns of ECM protein components and aberrant protease activity (Luibl et al., 2006; Jurkunas et al., 2009; Bhutto and Lutty, 2012; Okumura et al., 2015; Okumura et al., 2017; Ong Tone et al., 2021; Xu et al., 2021).

Proteases are known to play integral roles in nearly every physiological and pathological process. While human tissues express ∼600 different proteases, only a handful of human proteases have been extensively studied. One such well-studied protease is granzyme B (GrB). The normal and pathological functions of GrB have been investigated in a variety of organs and tissues and have implicated GrB in the process of tissue aging, as well as in a variety of age-related diseases (eg. cardiovascular disease, COPD, arthritis) (Hendel et al., 2010; Bao et al., 2018; Qiao et al., 2020; Zeglinski and Granville, 2020). However, little investigation has been conducted on GrB activity in ocular age-related diseases. The purpose of the current review is to discuss the putative role that GrB may play in the pathogenesis of AMD and FECD.

## Granzyme B in aging and age-related disease

### What is granzyme B

Granule-secreted enzymes (Granzymes) are a family of serine proteases that are evolutionarily conserved across a range of mammalian species (Plasman et al., 2013; Fu et al., 2016). The human genome encodes five different granzymes: granzyme A (a tryptase), granzyme B (an aspartase), granzyme H (a chymase), granzyme K (a tryptase) and granzyme M (a met-ase) (Grossman et al., 2003). With respect to biological function, GrB is the most widely studied and best described human granzyme (Trapani, 2012; Turner et al., 2019). GrB is encoded by the gene *GZMB,* which is located on chromosome 14 and consists of four introns and five exons (Klein et al., 1989). GrB itself is a 247 amino acid poly-peptide composed of three trans-domain segments and two 6-stranded β sheets (Estébanez-Perpiñá et al., 2000). A variety of immune and non-immune cells have been shown to express GrB (Table 1), and both intracellular and extracellular functions have been ascribed to this protease.

**TABLE 1:** Cell types that express Granzyme B.

List of cell types that have been shown to express granzyme B	
Immune cells	Non-immune cells
Cytotoxic T Lymphocytes and Natural Killer cells Canaday et al. (2001); Kelso et al. (2002)	Keratinocytes Hernandez-Pigeon et al. (2006)
CD4^+^ T cells Canaday et al. (2001); Hoek et al. (2021); Kawamura et al. (2006); Namekawa et al. (1998)	Type II pneumocytes Vernooy et al. (2007)
γδ T cells O’Neill et al. (2020); Ryan et al. (2016)	Retinal Pigment Epithelial Cells Matsubara et al. (2020a)
Mast cells Pardo et al. (2007); Strik et al. (2007)	Platelets Freishtat et al. (2009)
Basophils Tschopp et al. (2006)	Granulosa and Sertoli cells Sasson et al. (2003); Sipione et al. (2006)
Neutrophils Wagner et al. (2004)	Syncytial Trophoblasts Hirst et al. (2001)
Monocytes Elavazhagan et al. (2015)	Chondrocytes Horiuchi et al. (2003)
Macrophages Choy et al. (2003)	Mesenchymal Stromal cells Schüll et al. (2013)
Dendritic cells (both plasmacytoid and monocyte-derived) Korthals et al. (2007), Rissoan et al. (2002)	
B cells Hagn et al. (2009)	

Table of cell types validated to express granzyme B.

The majority of GrB research has focused on its intracellular role in the induction of apoptosis through the granule-induced apoptotic pathway (Boivin et al., 2009). GrB is a critical component of the lytic granules released by cytotoxic T lymphocytes (CTL) and Natural Killer (NK) cells. These cell types form an immunological synapse with target cells and release lytic granules containing both GrB and perforin into the synapse. Perforin multimerizes into the target cell’s plasma membrane, creating a 5–20 nm diameter pore and facilitating endocytosis of GrB into target cells. GrB then escapes from the endosome into the cytoplasm through a perforin-dependent manner and acts intracellularly to induce apoptosis through multiple caspase-dependent and caspase-independent mechanisms (Keefe et al., 2005; Shi et al., 2005; Pipkin and Lieberman, 2007; Boivin et al., 2009). This immune-mediated intracellular function of GrB has been found to be critical for anti-viral and anti-cancer immunity (Figure 3A) (Trapani and Sutton, 2003).

Recently, it has been revealed that GrB also exerts extracellular functions in certain biological contexts and pathologies, with implications for extracellular matrix (ECM) remodeling. The intracellular activity of GrB is largely dependent on perforin (Kägi et al., 1994; Smyth et al., 2000; Keefe et al., 2005; Shi et al., 2005; Pipkin and Lieberman, 2007); however, of the cells found to express GrB to date (see Table 1), only CTLs, NK cells, CD4^+^ T cells, γδ T cells, neutrophils and keratinocytes have been found to express perforin and GrB concomitantly (Wagner et al., 2004; Hernandez-Pigeon et al., 2006; Osińska et al., 2014; O’Neill et al., 2020). Thus, many of the cell types identified to express GrB do not express perforin and/or do not form immunological synapses with target cells and these cells are believed to release GrB directly into the local ECM. Moreover, CTLs, NK cells, CD4^+^ T cells and keratinocytes have been found to express GrB independently of perforin in certain contexts, suggesting these cells excrete GrB into the extracellular space of tissues under certain circumstances (Isaaz et al., 1995; Hernandez-Pigeon et al., 2007; Prakash et al., 2009; Lin et al., 2014). Indeed, several groups have independently shown that GrB is present extracellularly in the ECM of tissues and in bodily fluids (Isaaz et al., 1995; Tak et al., 1999; Choy et al., 2004). Furthermore, many ECM proteins have been validated as substrates of GrB, as have various tight junctional proteins, cell adhesion proteins and proteoglycans (Table 2). It is now appreciated that GrB likely plays a role in impaired ECM remodeling, induction of endothelial permeability, impaired epithelial barrier function, scar formation and anoikis in a variety of physiological situations (as reviewed by Boivin et al. (2009), Turner et al. (2019), and Choy Jonathan et al. (2004). Despite its recognized presence in the extracellular space, no effective endogenous extracellular GrB inhibitors have been identified in humans. Thus, it appears that GrB activity in the extracellular space is minimally regulated, which may help explain why GrB is implicated in a number of diseased states (Choy et al., 2003; Ngan et al., 2009; Turner et al., 2019; Matsubara et al., 2020; Qiao et al., 2020).

**TABLE 2:** Substrates of granzyme B.

Proteins involved in cell-cell and cell-extracellular matrix adhesion	Proteoglycans in basement membrane	Other basement membrane components
Vitronectin Buzza et al. (2005)	Aggrecan Froelich et al. (1993)	Fibronectin Buzza et al. (2005); Choy et al. (2004)
Smooth muscle cell matrix Choy et al. (2004)	Biglycan Boivin et al. (2012)	Laminin Buzza et al. (2005)
Fibrinogen Buzza et al. (2008)	Cartilage proteoglycans Froelich et al. (1993)	Fibrillin-1 Chamberlain et al. (2010)
VE-cadherin Pardo et al. (2007); Shen et al. (2016)	Decorin Ang et al. (2011); Boivin et al. (2012); Hiebert et al. (2013a); Parkinson et al. (2015)	Collagen VII Russo et al. (2018)
E-cadherin Turner et al. (2021)	β-glycan Boivin et al. (2012)	Collagen XVII Russo et al. (2018)
ZO-1 Pardo et al. (2007); Santacruz, (2018)		Collagen IV Jensen et al. (2020)
JAM-A Santacruz, (2018)		
Occludin Matsubara et al. (2020)		
α6 integrin Hiroyasu et al. (2021)		
Desmoglein 1, Desmoglein 3 Turner et al. (2021)		

Table of cell-cell and cell-extracellular matrix adhesion proteins, as well as proteoglycans, which act as substrate for GrB.

### Granzyme B in non-ocular age-related disease

**Skin Aging:** GrB has been implicated in the changes in tissue organization and tissue integrity associated with aging of skin. Apolipoprotein E knockout (ApoE KO) mice demonstrate signs of accelerated skin aging, including increased hair loss, skin thinning and collagen disorganization. These mice also express increased levels of GrB in their skin (Hiebert et al., 2011). Interestingly, ApoE/GrB double-knockout (ApoE/GrB DKO) mice exhibit increased hair follicle density and delayed graying of hair compared to ApoE KO mice (Cruz et al., 2006). Furthermore, ApoE/GrB DKO mice also display increased collagen density in their skin compared to ApoE KO mice (Hiebert et al., 2011). These findings suggest that GrB plays a role in age-related hair follicle attrition, as well as in age-related skin thinning and disorganization of dermal collagen. Finally, evidence suggests that GrB may contribute to the acceleration of skin aging seen with chronic ultraviolet light (UV) radiation exposure as well. UV radiation is widely considered to be a major factor in skin aging, inducing increased inflammation and enhanced ECM degradation. Interestingly, both UVA and UVB irradiation has been found to significantly increase GrB expression in keratinocytes in a redox-dependent manner (Hernandez-Pigeon et al., 2006; Hernandez-Pigeon et al., 2007; Parkinson et al., 2015). Under combined UVA and UVB irradiation (designed to reflect similar ratios to sunlight), GrB KO mice displayed reduced wrinkle formation and improved preservation of dermal collagen density compared to wild-type controls, which was attributed to the inhibition of decorin cleavage (Parkinson et al., 2015). Taken together, these findings suggest that GrB plays an appreciable role in the alterations in skin tissue associated with aging and photoaging.

**Cardiovascular Disease:** Evidence implicating GrB in CVD has been thoroughly summarized elsewhere (Zeglinski and Granville, 2020). In short, GrB levels are elevated in the plasma and relevant cardiovascular tissues of patients suffering from various CVDs (Kuijpers et al., 2003; Skjelland et al., 2007; Tsuru et al., 2008; Ikemoto et al., 2009; Kondo et al., 2009; Chamberlain et al., 2010). In a murine model of cardiac fibrosis, GrB deficiency significantly reduced interstitial and perivascular fibrosis, vascular permeability, and the number of activated myocardial fibroblasts, suggesting that GrB mediates several mechanisms underlying cardiac fibrosis (Shen et al., 2016). In murine models of aortic aneurysm, deficiency in GrB activity was found to reduce cleavage of ECM proteins decorin and fibrillin-1, increase microfibril structural integrity and improved collagen organization, leading to decreased aneurysm rupture and mortality (Chamberlain et al., 2010; Ang et al., 2011). These findings suggest that GrB promotes aneurysm formation through the degradation of key ECM proteins, reducing vessel integrity. In ApoE KO mice, GrB deficiency significantly increased longevity and reduced the formation of atherosclerotic plaques along the aorta, as well as improved collagen density/organization, suggesting GrB plays an influential role in plaque development and stability (Cruz et al., 2006; Hiebert et al., 2013). Finally, in a murine model of ischemic cerebral vascular disease, GrB inhibition reduced neuronal cell death and infarct volume, as well as improved both cognitive and motor functioning scores (Aslam and Yuan., 2020).

It is clear that GrB plays a role in the pathogenesis of various age-related disease, as outlined above, and it is likely that the role of GrB in other age-related diseases has yet to be elucidated.

## Age-related diseases in the eye

### Age-related macular degeneration

AMD is a multifactorial disease in which aging, genetics, and environment all play roles. Smoking habits and diet are well established AMD risk factors, and various genetic predispositions are also associated with the risk of developing AMD including common AMD-related variants in or near genes C2/CFB, C3, C9, CFH, CFI, ARMS2 and VTN and various rare variants in genes CFH, CFI, C3, C9 and TIMP3. These variants have helped to identify various pathways related to AMD pathogenesis with the central and most-understood pathway being the complement cascade pathway (Fritsche et al., 2016; Geerlings et al., 2017; Connolly et al., 2018; Colijn et al., 2021).

While considered a single disease, the early stage AMD may eventually lead to degenerative or proliferative forms of AMD (Jager et al., 2008; Ambati et al., 2013). The more common ‘dry’ (degenerative) form accounts for over 90% of AMD cases and leads to vision loss by the slow atrophy and death of retinal pigment epithelium (RPE), followed by photoreceptor (PR) loss in late-stage geographic atrophy (GA). The ‘wet’ (proliferative) form, which afflicts only 10% of patients, surprisingly causes over 90% of cases of visual impairment due to choroidal neovascularization (CNV), which leaks blood into the sensory retina and results in severe vision loss. Current standard of care for CNV includes intravitreal injections of biologics (e.g: Ranibizumab, Bevacizumab, Aflibercept) that target vascular endothelial growth factor (VEGF), a key mediator of CNV. However, this treatment is suboptimal, as up to 25% of patients are non-responsive to anti-VEGFs while others develop drug tolerance (Ehlken et al., 2014; Tanaka et al., 2015; Yang et al., 2016; Grunwald et al., 2017). One essential event in early-stage AMD that provokes the development of both dry and wet forms is the breakdown of Bruch’s membrane (BrM), an important extracellular matrix. BrM is the substratum of the RPE, choroidal endothelial cells and blood vessel walls, and undergoes significant remodeling during aging (Nita et al., 2014). The outer blood retinal barrier (oBRB) loses functionality when tight junctions between RPE cells degrade, triggering early stage AMD by promoting RPE dysfunction, inflammation and vascular permeability (Bhutto and Lutty, 2012). Features of a disrupted oBRB also promote wet and dry forms of AMD.

### Retinal pigment epithelium

Located between the metabolically active photoreceptors and the fenestrated choriocapillaris blood supply, the RPE is strategically positioned to maintain retinal homeostasis, regulating access to nutrients from the blood to the photoreceptors as well as eliminating waste products (Figures 1B–D, adapted from Matsubara et al., 2020) (Bhutto and Lutty, 2012; Nita et al., 2014). Other important roles include the absorption of scattered light, retinal adhesion, vitamin A transport and processing, and re-isomerization of all-trans-retinal to 11-cis retinal, which is crucial for the visual cycle (Naylor et al., 2019). Tight junctions between neighboring RPE cells strictly control the movement of fluid and solutes across the oBRB. The RPE selectively transports nutrients into the outer retina, as well as transports metabolic waste produced by the photoreceptors out of the outer retina into the choroidal vascular bed. The RPE also pumps water out of the retina to maintain the retina’s hydration status and facilitate attachment of the retina to the RPE. The RPE does this using ion pumps to transport ions from the subretinal space into the choroid, producing a net ion flux which forces the water to follow (Simó et al., 2010).

**FIGURE 2:**
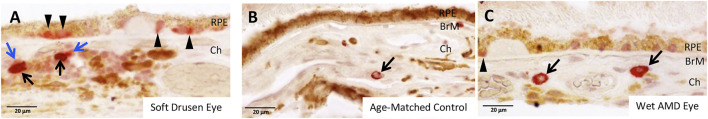
Retinal Pigment Epithelium (RPE) and Choroid (Ch) demonstrate stronger GrB immunoreactivity (red AEC, arrows and arrowheads) in soft drusen eyes **(A)** compared to age-matched control eyes **(B)**. GrB is also stronger in the choroid of wet AMD eyes **(C)** compared to age-matched controls **(B)**. Blue arrows indicate degranulated mast cells in choroid. Bruch’s membrane (BrM). Adapted from Matsubara et al., 2020.

Recently we showed by immunohistochemistry that GrB accumulates with age, and significantly in eyes that display the wet form of AMD. GrB is present in mast cells in the choroid, and in the basal compartment of the RPE cell in human tissues (Matsubara et al., 2020). A comparison of GrB distribution in eyes with dry AMD, wet AMD or soft drusen (a hallmark of early‐stage AMD) revealed that the choroidal mast cells in wet AMD eyes displayed the strongest GrB immunolabeling compared to dry AMD eyes or eyes with soft drusen, a hallmark of early-stage AMD. However, eyes with soft drusen displayed the strongest GrB immunolabeling in the RPE cell compared to the wet or dry AMD groups (Figure 2, adapted from Matsubara et al., 2020). This suggests that GrB in the RPE cell may contribute to early stages of AMD development, while GrB in choroidal mast cells may contribute to wet AMD. In aging rodent models, extracellular GrB is present in BM and in the intercellular spaces between the RPE. Both BM and the intercellular spaces between RPE contain relevant substrates of GrB including fibronectin, laminins, tight junctional complexes (ZO-1) and junctional adhesion proteins (JAM). RPE culture experiments demonstrated that exogenous GrB cleaved RPE-derived ZO-1, JAM-A, FN and LAM (Matsubara et al., 2020). These *in vitro* studies point to the putative role of GrB in AMD, where the breakdown of the oBRB is an early event in the pathogenesis of AMD, and specifically in the development of CNV in the wet form of AMD.

### Fuchs endothelial corneal dystrophy

FECD is a multifaceted corneal endothelial dystrophy and is the leading non-iatrogenic indication for corneal transplantation worldwide (Gain et al., 2016; Ong Tone et al., 2021). FECD clinically manifests as exaggerated CE density loss, prominent CEC polymegathism and pleomorphism, abnormal thickening of Descemet’s membrane (DM) and the formation of protein deposits called guttae, all of which culminate in corneal edema and loss of corneal transparency. Thickening of the DM is driven by increased ECM protein secretion and altered ECM component arrangement. Guttae are excrescences of DM and its ECM proteins which appear under discrete regions of the CE (Figures 1E–G) (Elhalis et al., 2010; Weller et al., 2014; Xia et al., 2016; Hribek et al., 2021). FECD pathophysiology begins in the central CE and spreads peripherally as the disease progresses. Risk factors for FECD include age, female gender, prolonged UV exposure and smoking (Ong Tone et al., 2021). Genetically, FECD is a complex and heterogeneous disease with dozens of causal mutations identified to date. Early-onset FECD has been ascribed to mutations in the genes COL8A2, TCF4, TCF8, SLC4A11 and AGBL1, while late-onset FECD, which is far more common than early-onset FECD, is a sporadic disease (Liu et al., 2021). Despite this, the clinical phenotype of FECD varies little in its manifestation and progression. The major pathological features of FECD are oxidative stress and increased ROS production, reduced antioxidant expression, mitochondrial dysfunction, DNA damage, ER stress and abnormal ECM deposition. A thorough review of FECD can be found in Ong Tone et al. (2021).

### Corneal endothelium

The corneal endothelium (CE) is a monolayer of post-mitotic polygonal cells of neuroectoderm origin (Katikireddy et al., 2016) and functions as a semi-permeable membrane and ion pump. The CE keeps the cornea transparent by maintaining deturgescence (relative dehydrated state) within the corneal stroma. Stromal deturgescence is regulated mainly by the CE which allows nutrient passage from the aqueous humor into the cornea, but at the same time, limits the accumulation of water into the stroma. The CE accomplishes this through limited permeability to water through actively pumping ions into the aqueous humor, which produces an osmotic force that pulls stromal water back into the aqueous humor (Barfort and Maurice, 1974; Bonanno, 2003).

Another function of the CE is to sustain DM. DM is a basement membrane composed of multiple ECM proteins, including fibronectin, laminins, collagen types I, IV and VIII, and various proteoglycans (LeBleu et al., 2007). The DM is composed of two distinct layers. The anterior layer of the DM is known as the Anterior Banded Layer (ABL) and is fully formed by birth. The Posterior Non-Banded Layer (PNBL) is not present at birth and is continuously laid down by ECM excrescence from CE over the lifespan of an individual (Murphy et al., 1984). The PNBL thickens by ∼ 0.01 μm/year (Reim, 1984). In FECD, aberrant ECM production leads to the formation of an additional structurally distinct third layer in DM, the Posterior Banded Layer (PBL). The PBL is posterior to the PNBL and is composed largely of abnormally deposited collagen and guttae (Elhalis et al., 2010).

Preliminary immunohistochemistry performed on human post-mortem corneas found an age-related increase in GrB in the CE of the control eyes and in eyes with FECD compared to control (unpublished data). The presence of GrB in the CE of control and FECD afflicted corneas in this prefatory work suggests that GrB may have a role in the CE in both normal CE health and in FECD. More work is required to elucidate the significance of GrB activity in the CE.

### Similarities between retinal pigment epithelium and corneal endothelium

Interestingly, the CE has embryonic, structural, and functional similarities to the RPE. Both tissues originate from the neuroectoderm (Fuhrmann et al., 2014). Furthermore, they have structural similarities, as both are monolayers of post-mitotic polygonal cells attached to thick basement membranes (Fuhrmann et al., 2014). Lastly, both tissues act as semi-permeable barriers and ion pumps, expressing tight junctional proteins which are integral to their functions (Figures 1D,G).

The similarities between the CE and RPE also extend to the most common degenerative diseases that afflict these tissues. Both AMD and FECD are characterized by cellular stress and degeneration of a post-mitotic monolayer (the RPE in AMD and the CE in FECD), reduced monolayer barrier integrity (breakdown of the oBRB in AMD and of the CE in FECD), as well as disorganization of the local ECM with accumulation of protein deposits on the underlying basement membrane (altered ECM protein composition and drusen accumulation in BrM in AMD and altered ECM protein composition and guttae accumulation in the DM in FECD (Figures 1C,F). AMD and FECD also possess similar risk factors, including age, female gender, and exposure to environmental stressors such as smoking and UV radiation (Jager et al., 2008; Zhang et al., 2013; Liu et al., 2020). Oxidative stress has also been strongly implicated in the development of both AMD and FECD (Jurkunas, 2018; Ong Tone et al., 2021). Additionally, a prospective study found an increased prevalence of AMD in FECD patients compared to controls without any corneal pathology (Rao et al., 1998). This suggests that the presence of FECD pathogenic markers may imply an increased risk of AMD. This was further corroborated by a recent cross-sectional study done by Shi et al., 2022, which found a positive correlation between the degree of FECD severity and the presence of AMD. However, a separate cross-sectional study done by Matthaei et al., 2018, found no correlation between the amount of macular drusen and FECD. The diverging outcomes of these studies might be related to different sample populations and grading methodologies. Regardless, the similarities between FECD and AMD suggest that these diseases may share common underlying pathogenic mechanisms that go beyond oxidative stress.

### Potential contributions of granzyme B to AMD

Although AMD is a complex multifactorial disease, inflammatory processes, including leukocyte infiltration and activation, and cytokine upregulation, critically contribute to the early phase and the progression of CNV in wet AMD. The upregulation of various proinflammatory cytokines, including IL-6, TGF-β, IL-1β, TNF-α and proangiogenic factors including VEGF, platelet-derived growth factor (PDGF), placental growth factor (PlGF), fibroblast growth factor-2 (FGF-2) promote chronic low-grade inflammation, causing enhancement of vascular permeability and elevation of proangiogenic responses in choroidal endothelial cells (Amin et al., 1994; Rakic et al., 2003; Jo et al., 2006; Browning et al., 2008; Nourinia et al., 2013; Chen and Xu, 2015; Kauppinen et al., 2016). Therefore, investigating the mechanisms of inflammation is critical to the therapeutic management of CNV (Chen and Xu, 2015; Kauppinen et al., 2016).

The BrM provides barrier, support and transport functions to the RPE, while the choroidal vascular wall ECM provides structural support to the vascular endothelium and regulates various angiogenesis, proliferation and survival signalling pathways (Davis and Senger, 2005; Booij et al., 2010; Lu et al., 2011). Alterations to these ECMs can cause significant dysfunction. Indeed, disruption of the BrM, possibly due to the formation of soft drusen, is an early trigger for the development of AMD (Chong et al., 2005; Nita et al., 2014; van Lookeren Campagne et al., 2014). Furthermore, in response to aging and disease, the choroidal vascular wall ECM and cell tight junctions which regulate vascular permeability in quiescent cells degrade, promoting initiation of angiogenesis and enhancement of vascular permeability (Berglin et al., 2003; Alghisi et al., 2009; Nita et al., 2014; Mansoor et al., 2019).

Wet AMD is characterised by CNV (which can disrupt the BrM), chronic inflammation, and vascular permeability (Chong et al., 2005; van Lookeren Campagne et al., 2014). Extracellular GrB in the choroid and RPE layers is mainly sourced from overactive, degranulating choroidal mast cells (Matsubara et al., 2020). Mast cells are critical mediators of ocular inflammation, and RPE anomalies and oBrB breakdown were observed in rodents after experimental degranulation of choroidal mast cells (Sebastião da Silva et al., 2011; Bousquet et al., 2015). In the oBRB, initiation of ECM and RPE tight junction degradation by the cleavage activity of extracellular GrB is a potential initiator of the aforementioned pathologic characteristics of AMD, which is supported by earlier *in vitro* studies (Matsubara et al., 2020). The GrB-mediated cleavage of ECM proteins can potentially initiate and sustain the inflammation that progresses to CNV through different pathways including: 1) Increased chemotaxis facilitated by the cleavage fragments of certain ECM and tight junction proteins e.g., fibronectin fragments (FN-fs) (Parkinson et al., 2015), which fosters inflammation by recruiting leukocytes and promoting MMP activity and release of IL-6, IL-8, TNF-α and CCL2 (Pulai et al., 2005; Marom et al., 2007; Austin et al., 2009; Chen and Xu, 2015; Hiroyasu et al., 2021); 2) Increased processing of proinflammatory cytokine such as IL-6, IL-8 and TGF-β, which can stimulate further cytokine production and upregulate choroidal endothelial cell proliferation (Iruela-Arispe and Sage, 1993; Nagineni et al., 2003; Izumi-Nagai et al., 2007; Gao et al., 2021; Hiroyasu et al., 2021); indeed, GrB has been shown to induce IL-8 expression in keratinocytes and also release decorin-sequestered active TGF-β from proteoglycans (Boivin et al., 2012; Hiroyasu et al., 2021); 3) Increased extracellular pro-angiogenic factors including VEGF-A and reduced antiangiogenic factors including thrombospondin-1 (TSP-1), which further establishes CNV and vascular permeability (Kwak et al., 2000; Uno et al., 2006; Bhutto et al., 2008; Wang et al., 2012; Hendel et al., 2014; Li et al., 2020); this pathway is supported by data from Hendel et al. (2014) which showed that GrB mediates the release of VEGF from fibronectin, increasing vascular permeability; and 4) The promotion of cell death (PCD) by GrB-induced ECM detachment (anoikis) to release damage-associated molecular pattern (DAMP) molecules or alarmins from the ECM, inducing immune responses which can further sustain inflammation (Gilmore, 2005; Ghiringhelli et al., 2009; Dupont et al., 2011; Petrovski et al., 2011; Nita et al., 2014; Szatmári-Tóth et al., 2019)**.**

In a recent unpublished study, we showed that in an *ex-vivo* model of choroidal angiogenesis, the choroid sprouting assay (CSA), exogenous GrB can potentially contribute to choroidal neovascularization through ECM remodelling, pro-inflammatory and pro-angiogenesis pathways (Obasanmi et al., 2021). In this model, treatment with exogenous GrB results in significantly larger area of choroidal microvascular angiogenesis compared with controls. Exogenous GrB stimulation also yielded ECM degradation, including increased cleavage of fibronectin, laminin and decorin, along with increased expression of proinflammatory/proangiogenic factors, such as IL-6, TGF-B and VEGF, in supernatants of the RPE-choroid-sclera *ex-vivo* culture. Furthermore, the combination of these activities of GrB can weaken the function of the oBRB, initiating and/or accelerating inflammation, vascular permeability, and pathological choroidal neovascularization (Hendel et al., 2014).

**FIGURE 3:**
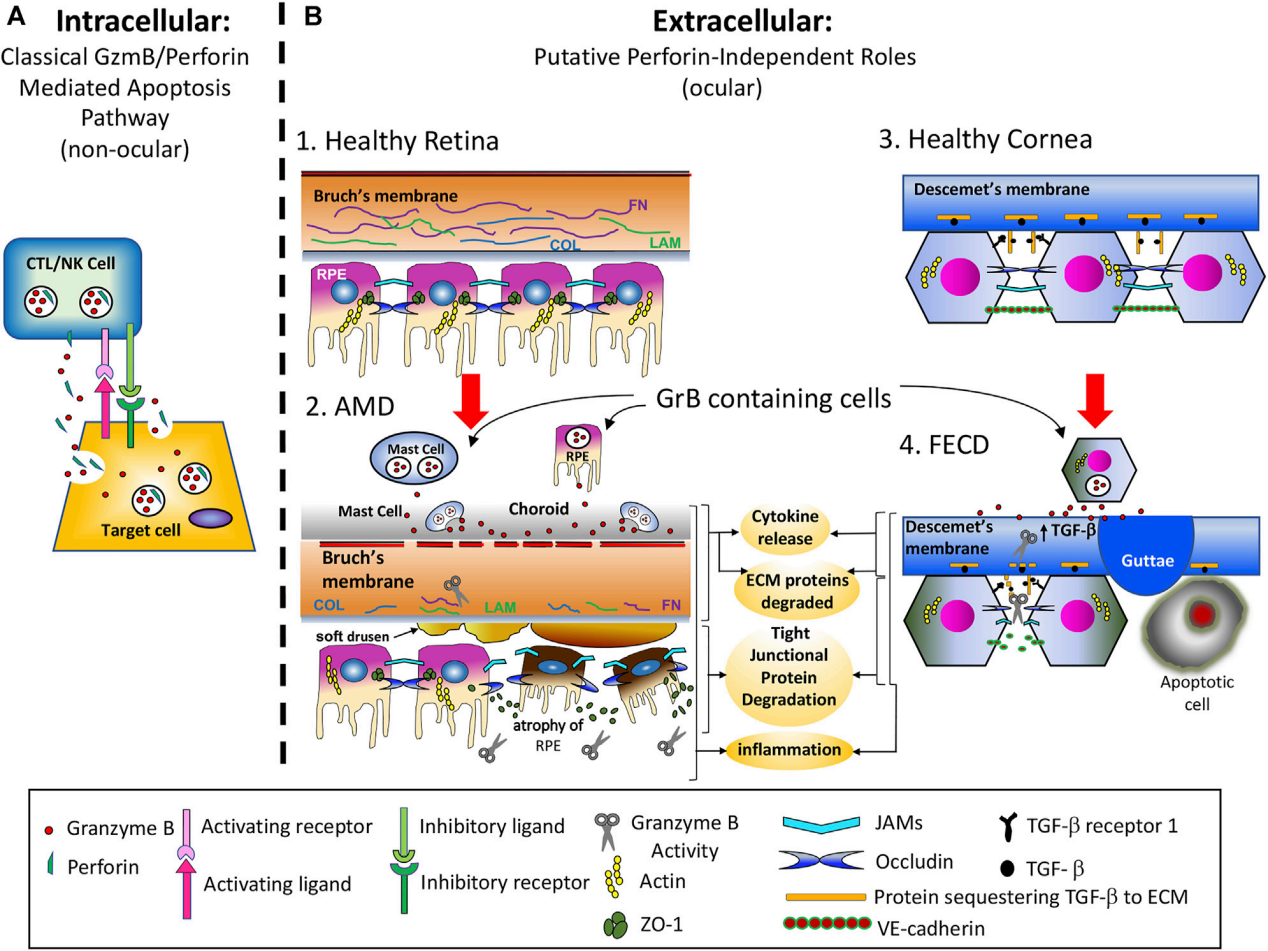
Intracellular vs. Extracellular Functions of GrB. **(A)**. GrB’s intracellular role is in the induction of apoptosis through the granule-induced apoptotic pathway. GrB is a critical component of the lytic granules released by cytotoxic T lymphocytes (CTL) and Natural Killer (NK) cells. These cell types form an immunological synapse with target cells and release lytic granules containing both GrB and perforin into the synapse. Perforin multimerizes into the target cell’s plasma membrane, creating a 5–20 nm diameter pore and facilitates endocytosis of GrB into target cells, wherein it then escapes from the endosome into the cytoplasm through a perforin-dependent manner and acts intracellularly to induce apoptosis through multiple caspase-dependent and caspase-independent mechanisms. **(B)**. GrB’s extracellular role is in the promoting cytokine release, extracellular matrix (ECM) protein cleavage, tight junctional (TJ) protein degradation, and proinflammation in AMD and FECD. B1) Outer retina of a healthy eye with intact Bruch’s membrane and RPE barrier functions in the absence of extracellular GrB activity. B2) Age-related buildup of extracellular GrB degrades ECM and Ti proteins. Known substrates of GrB include FN, LAM, COL, JAMS and occludin. The early pathological sequence of AMD development (before vision loss) include: i) ECM remodeling and pro-inflammation; ii) Accumulation of lipoproteinaceous material (including soft drusen deposits) between Bruch’s membrane and RPE; iii) RPE gene dysregulation, atrophy and cell death associated with the dry form of AMD; iv) While it is not known why or how dry AMD transitions to wet AMD, it is known that in most cases, the dry form precedes the wet form of AMD. B3) Descemet’s membrane and corneal endothelium of a healthy eye with intact corneal endothelial barrier function. No extracellular GrB is present. B4) Descemet’s membrane and corneal endothelium of an eye with FECD. The putative sequence of events that lead to FECD include: i) the corneal endothelium becomes increasingly permeable to water, TGF-β signaling is upregulated, causing increased ECM protein production, and corneal endothelial cells (CECs) accumulate both ER stress and oxidative stress; ii) Descemet’s membrane (DM) becomes abnormally thick and cytotoxic protein deposits called guttoe form, iii) CECs undergo polymegathism, pleomorphism and ultimately apoptosis.

We speculate that aberrant GrB present within the extracellular space cleaves ECMs such as FN, LAM, and COL which lead to a disorganized Bruch’s membrane, and degrades tight junctional proteins (JAMs and occludin) between RPE cells thereby disrupting the outer blood eye barrier, a key event in choroidal neovascularization in wet AMD. Cleavage fragments of these substrates can increase chemotaxis, thereby promoting inflammation and increasing proinflammatory cytokines such as IL-6, TGF-β, TNF-α and CCL2 which can stimulate choroidal endothelial cell proliferation. Furthermore, extracellular GrB can directly or indirectly increase pro-angiogenesis by increasing proangiogenic VEGF-A expression while cleaving antiangiogenic TSP-1. The presence of soft drusen that separates the RPE from the BrM, ECM fragility, proinflammation and pro-angiogenesis can render the subretinal space vulnerable to pathological insults and vascular invasion, promote cell death and sustain chronic inflammation, thereby advancing wet and dry forms of AMD (Figure 3B). The pharmacological inhibition of extracellular GrB in the chorio-retinal space may be a potentially therapeutic option that can correct the pathologic ECM remodelling induced by GrB, thereby restraining its concomitant effects and the pathologic characteristics of CNV in AMD.

### Potential contributions of granzyme B to FECD

Given the similar characteristics described earlier between FECD and AMD, GrB may also contribute to FECD pathophysiology as well. It is important to note that in AMD, over-active mast cells within the choroid represent a major source of GrB within both the choroid and RPE layers, while FECD is primarily a non-inflammatory disorder (Pardo et al., 2007; Parkinson et al., 2015; Wroblewski et al., 2017; Matsubara et al., 2020; Hiroyasu et al., 2021). Therefore, CECs are the likely source of GrB in FECD. This abnormal extracellular GrB activity in FECD may be contributing disease pathogenesis through facilitation of aberrant TGF-β signaling and impaired CE barrier integrity.

Excessive ECM production and ER stress are prominent features of FECD. Aberrant TGF-β signaling is strongly implicated in these pathological processes. TGF-β is a multifaceted signaling molecule that is constitutively present in the ECM of most tissues in a latent form, where it is sequestered in ECM by TGF-β reservoirs such as the proteoglycans decorin, biglycan and β-glycan (Boivin et al., 2009). TGF-β signaling mediates many CEC processes, including quiescence, tight junction formation, the production of various ECM proteins and endothelial-to-mesenchymal transition (Roberts et al., 1992; Joyce et al., 2002; Verrecchia and Mauviel, 2002; Li et al., 2013; Beaulieu Leclerc et al., 2018). TGF-β signaling regulates the expression of many ECM components, such as collagens type I and III, keratoepithelin/TGFBI, clusterin and fibronectin (Zhang et al., 1995; Hocevar et al., 1999; Thapa et al., 2007; Shiota et al., 2012; Pan et al., 2013). FECD-CECs are found to overexpress TGF-β1, TGF-β2, and TGF-β receptor 1, and the PBL and guttae, which are abnormal FECD-related DM structures, appear to be composed of many of these TGF-β-regulated ECM components (Jurkunas et al., 2009; Weller et al., 2014; Xia et al., 2016; Okumura et al., 2017). Furthermore, excessive TGF-β is known to precipitate ER stress in CECs (Okumura et al., 2017, 2015). Inhibition of TGF-β signaling suppressed aberrant ECM production, guttae formation and the accumulation of unfolded proteins in FECD-CECs (Okumura et al., 2017, 2015).

Given its role in FECD pathogenesis, TGF-β signaling presents an enticing therapeutic target to slow or inhibit FECD progression. Unfortunately, due to the variety of crucial CEC processes regulated by TGF-β signaling, therapeutic inhibition TGF-β signaling without significant impairment of CE function may prove difficult. However, release of TGF-β can occur through multiple distinct mechanisms (Gleizes et al., 1997; Jobling et al., 2006; Taylor, 2009; Boivin et al., 2012). Targeting mechanisms of TGF-β-activation upregulated in FECD may provide a more viable therapeutic strategy to reduce pathological TGF-β-activation while preserving sufficient TGF-β signaling for proper CE function. Interestingly, ROS alone have been shown to release TGF-β from the ECM and amplify TGF-β-mediated signal transduction (Jobling et al., 2006; Jain et al., 2013). Furthermore, treatment with N-acetylcysteine, a thiol-containing antioxidant and radical scavenger, reduced guttae formation, decreased markers of ER stress and significantly improved corneal density in murine models of FECD, suggesting ROS play a role in TGF-β-mediated pathology in FECD (Kim et al., 2014).

GrB and other proteases, such as MMPs, represent another mechanism of TGF-β release (Taylor, 2009; Boivin et al., 2012). CECs express multiple MMPs, including MMPs 2, three and 7. MMP 2, three and seven have been shown to cleave either decorin, biglycan or beta-glycan to release TGF-β (Imai et al., 1997; Xu et al., 2021). However, in FECD, MMP-2 expression is downregulated and the expression of various MMP inhibitors (TIMPs) are upregulated (Xu et al., 2021). Consequently, it seems less likely that aberrant MMP activity is contributing significantly to the increased TGF-β signaling in FECD. In contrast, preliminary work has shown that GrB can be found within the CE in FECD (unpublished data). GrB can release TGF-β from many putative TGF-β reservoirs found in the CE/basal DM, including decorin, biglycan, β-glycan and fibrillin-1, and is the only protease known to cleave each of these substrates (Leung et al., 2000; Schlötzer-Schrehardt et al., 2011; Poulsen et al., 2014). Furthermore, GrB has no endogenous identified extracellular inhibitor (Chamberlain et al., 2010), suggesting that excessive extracellular GrB can efficiently activate TGF-β and significantly amplify local TGF-β signaling. Moreover, unlike MMPs, initial immunohistochemistry suggests that GrB is only weakly present in non-elderly healthy CE (unpublished data), implying that GrB may not be critical for rudimentary CE homeostasis. Therefore, GrB inhibition may be less likely to significantly impair normal CEC function compared to inhibition of MMPs. While future work is required to further elucidate the potential role for GrB in healthy CE and in FECD, inhibition of extracellular GrB activity may represent a potential therapy to reduce aberrant TGF- β signaling. Furthermore, its combination with antioxidants may function synergistically to reduce ROS production and significantly dampen the pathological mechanisms underlying aberrant TGF-β signaling in FECD, potentially rescuing normal TGF-β signaling.

Aberrant GrB activity in the local extracellular space may also be contributing to impaired barrier integrity in FECD through degradation of ECM components, including cell-cell contacts, as it does in AMD. CE barrier integrity appears compromised in the early stages of FECD, even before clinical evidence of corneal edema is present (Burns et al., 1981). CE barrier integrity is contingent on both tight junctions and adherens junctions. Not much research has focused on tight junctions and adherens junction in the CE, though they are believed to express many of the typical tight junction and adherens junction proteins (Figure 1). Various tight junction and adherens junction components act as substrates for GrB, including ZO-1, JAM-A, occludins and VE-cadherin (Table 2). Levels of ZO-1 are not reduced in FECD, though ZO-1 organization is impaired (Chalimeswamy et al., 2022). Interestingly, loss of JAM-A expression has been shown to impair ZO-1 organization and cell-cell interactions (Otani et al., 2019). Furthermore, loss of CEC JAM-A interactions alone appears sufficient for corneal edema to ensue (Mandell et al., 2006). Additionally, disruption of VE-cadherin has been shown to increase vascular endothelial permeability and loss of GrB expression was found to reduce VE-cadherin cleavage and vascular leakage in murine models of cardiac fibrosis (Roh et al., 2013; Shen et al., 2016). As JAM-A and VE-cadherin are expressed extracellularly by CECs and are substrates of GrB, these finding suggest a potential role for aberrant extracellular GrB activity in the reduction of CE barrier integrity in FECD.

We speculate that aberrant GrB is present within the extracellular space in FECD, contributing to an increase in the release of TGF-β from many of its ECM reservoirs, including decorin, biglycan, β-glycan and fibrillin-1, into the local ECM. Increased TGF-β signaling, in turn, results in significant upregulation of protein synthesis, the induction of the ER stress response as well as further accumulation of oxidative stress, and the formation of cytotoxic guttae, leading to CEC pleomorphism, polymegathism and apoptosis. Furthermore, aberrant GrB activity in the extracellular space results in cleavage of adhesion junction proteins and disorganization of tight junction proteins, resulting in increased corneal permeability and leading to corneal edema (Figure 3B). Ultimately, more work is needed to investigate the potential role of GrB in CE barrier dysfunction in FECD. Nevertheless, GrB inhibition may represent therapeutic avenue to help maintain barrier integrity in FECD.

### Current therapeutic strategies to modulate extracellular GrB activity

Evidence for targeting extracellular GrB has been validated via models of various cardiovascular, dermatological, neuroinflammatory, gastroenterological and respiratory pathologies utilizing genetic knockouts, biologics and/or small molecules (Aneurysm - (Chamberlain et al., 2010; Ang et al., 2011); Atherosclerosis - (Paul R. Hiebert PR. et al., 2013); Cardiac fibrosis - (Shen et al., 2016); Cerebral ischemia - (Aslam and Yuan, 2020); Vascular leakage -(Hendel and Granville, 2013; Hendel et al., 2014); Chronic wound healing - (P. R. Hiebert P. R. et al., 2013; Hsu et al., 2014; Turner et al., 2021a); Skin aging - (Hiebert et al., 2011; Parkinson et al., 2015); Scarring - (Shen et al., 2018); Autoimmune blistering - (Russo et al., 2018; Hiroyasu et al., 2021); Dermatitis - (Turner et al., 2021); Multiple sclerosis - (Haile et al., 2015); Crohn’s disease - (Hu et al., 2022); Asthma - (Qian et al., 2020)). From these studies, pharmacological inhibition of extracellular GrB appears to be the most viable avenue of therapy, with the biologic Serpina3n and the small molecule VTI-1002 both exhibiting therapeutic efficacy.

Serpina3n is a murine serine protease inhibitor homologous to the human α1-antichymotrypsin protein. Both Serpina3n and α1-antichymotrypsin can target and inactivate a multitude of proteases; however, unlike α1-antichymotrypsin, SerpinA3n is able to bind to and inhibit extracellular GrB (Sipione et al., 2006; Aslam et al., 2022). In murine model of Aortic Aneurysms, systemic Serpina3n treatment was shown to reduce degradation of the proteoglycan decorin while increasing collagen density the aorta of mice, leading to a reduced frequency of aortic rupture and death (Ang et al., 2011). In a murine model of MS, systemic Serpina3n treatment exhibited neuroprotective effects, reducing neuronal death, axonal injury and helping to maintain myelin integrity (Haile et al., 2015). Systemic treatment with Serpina3n also reduced the volume of cerebral infarct and improved neurological function in a murine model of ischemic stroke (Aslam and Yuan, 2020). Despite evidence of its efficacy in certain pathologies, the low specificity of Serpina3n is a limitation. Aside from GrB, Serpina3n has also been found to target anti-trypsin, anti-chymotrypsin, cathepsin G, elastase, MMP9, and selective cysteine proteases (Gettins, 2002; Horvath et al., 2005). This promiscuity poses the risk of off-target effects and unwanted side effects, especially in the context of regular use in chronic diseases. Furthermore, safety of repeated Serpina3n treatments remains unclear, as the studies exploring the efficacy of Serpina3n all used single dose treatment regiments. As such, the viability of regular Serpina3n treatment as part of chronic disease management has yet to be elucidated.

VTI-1002 is a small molecule developed by viDA Therapeutics (Vancouver, BC). VTI-1002 is a highly potent (Ki∼4.4 nM) inhibitor of human GrB and murine GrB (IC50 = 179 nM) and is highly specific, exhibiting minimal inhibition against other proteases including neutrophil elastase, cathepsin G, and caspases 3,4,5,6,7,8,9, and 10 (IC50 > 300uM) (Shen et al., 2018; Jung et al., 2022). Gel-formulated VTI-1002 is retained in skin for up to 24 h with minimal systemic absorption and no adverse events observed over 30 days of daily topical administration In a murine model of diabetic burn wound healing, VTI-1002 reduced scarring while improving collagen remodeling and tensile strength through a process involving the inhibition of GrB-mediated cleavage of decorin (Shen et al., 2018). In a murine model of atopic dermatitis, topical VTI-1002 reduced loss of epithelial barrier function and disease severity through prevention of E-cadherin and filaggrin cleavage (Turner et al., 2021). Lastly, VTI-1002 was found to prevent the cleavage of key hemidesmosomal proteins within the basement membrane including collagen XVII, α6β4 integrin as well as collagen VII in murine models of pemphigoid diseases (Hiroyasu et al., 2021). Given its high affinity and specificity for GrB with no evidence of adverse effects following repeated treatments in *vivo* models, VTI-1002 demonstrates potential as an anti-GrB therapy.

### Putative Anti-GrB therapies for AMD and FECD

Medical management of wet-AMD requires delivery of therapeutic agents to the retina. Systemic administration methods are the least invasive route of drug delivery; however, due to the outer and inner blood-retina barrier, the influx of a drug into the retina is limited. Thus, systemic administration would require very high doses to achieve therapeutic concentrations within the retina, and would likely lead to side effects or even toxicity in other tissues (Myles et al., 2005; Awwad et al., 2017). Therefore, local administration of retinal therapies is necessary. Intravitreal injection is the current standard for drug administration to the retina (Awwad et al., 2017). Intravitreal injection of anti-VEFG biologics is the gold standard of medical management of wet-AMD, though up to 25% of patients are non-responsive to anti-VEGFs while others develop drug tolerance (Ehlken et al., 2014; Tanaka et al., 2015; Yang et al., 2016; Grunwald et al., 2017). Consequently, alternative treatments for wet-AMD are needed, and intravitreal injections of VTI-1002, either alone or in conjunction with anti-VEGF biologics (where they may exhibit synergistic effects on VEGF signaling), represent an exciting potential avenue for treatment of wet-AMD.

Other obstacles exist for drug delivery to the CE. The cornea is avascular. Thus, systemically administered drugs must pass through multiple selectively permeable barriers, including the blood–aqueous barrier, and diffuse through the aqueous humor to reach the cornea, making systemic drug delivery to the CE impractical (Barar et al., 2008). However, the superficial nature of the cornea makes it highly amenable to topical routes of administration. Unfortunately, the variable hydrophilicity of the corneal layers impacts the permeability of topical agents, while the ocular cul-de-sac limits the volume of drug solution able to be administrated and tear film turnover and lacrimation limit drug residence time. These physical and biochemical barriers are estimated to limit the bioavailability of a topically applied agent to ≤5% for some substances (Patel et al., 2013; Awwad et al., 2017; Jimenez et al., 2019). Nonetheless, topical agents targeting structures posterior to the cornea, such as those used in glaucoma and non-infectious anterior uveitis treatment, demonstrate that topical drug therapy can permeate the cornea and be used for anterior segment pathologies (Harthan et al., 2016; K. Schuster et al., 2020). Moreover, multiple strategies exist to help increase the ocular bioavailability of topical agents. Pro-drug formulations and *in-situ* processing can facilitate passage through the variable hydrophilic layers of the cornea, while techniques like iontophoresis can increase corneal permeability (Awwad et al., 2017; del Amo and Urtti, 2008; Digiuni et al., 2012). Furthermore, drug load can be increased through the use emulsions, while increasing viscosity using vehicles or ointments, or adding drug carriers to bind to the tear film, can help to overcome limitations from tear film turnover and lacrimation (as reviewed by Awwad et al., 2017). As such, a topical formulation of VTI-1002 suitable for ocular use to target the CE is feasible. Currently, no therapeutic agent exists to inhibit or hinder the progression of FECD. Given that aberrant extracellular GrB activity may be contributing to FECD pathogenesis, VTI-1002, either alone or in conjunction with antioxidant therapy may have the potential to attenuate the progression of FECD.

## Conclusion

Aberrant GrB activity in the retina and cornea may be significantly contributing to pathogenesis of common age-related diseases that effect these tissues in AMD and FECD.

In the absence of any known endogenous GrB inhibitor, the unrestrained cleavage activity of extracellular GrB in the chorio-retinal space may be central to the potential contributions of GrB to CNV in AMD pathophysiology through remodelling of the local ECM, leading to increasing pro-inflammaotry cytokine expression and release of pro-angiogenetic factors such as VEGF from the ECM. Therefore, the pharmacological inhibition of extracellular GrB as a potential alternative/adjuvant therapy to current mainstay anti-VEGF biologicals to treat CNV might rescue the potential accompanying pathological consequences of extracellular GrB activity, which include excessive inflammation and angiogenesis. Such therapy might bring relief to CNV patients who do not respond to mainstay treatments, need higher frequency of mainstay treatments, or have developed resistance to mainstay treatments. VTI-1002 is a potent and specific inhibitor of GrB and may potentially prove to be an efficacious therapeutic agent in the treatment of CNV in wet AMD.

In FECD, GrB has the potential to contribute to many pathological characteristics through increasing TGF-β and through cleavage of cell-adhesion proteins. TGF-β is vital for the maintenance of the CE and therefore may not be a viable therapeutic target (Beaulieu Leclerc et al., 2018; (Kubiczkova et al., 2012). However, targeting GrB in the CE represents a potential alternative to dampen excessive TGF-β-signaling. Furthermore, inhibiting GrB activity in the CE may also ameliorate barrier integrity in the early stages of FECD and reduce the cellular stress produced by compensatory upregulation of pump activity. In conjunction with antioxidant therapies, GrB-directed therapies, such as VTI-1002, have the potential to attenuate the pathogenesis of FECD.
